# Intracranial Atherosclerotic Disease-Related Acute Middle Cerebral Artery Occlusion Can Be Predicted by Diffusion-Weighted Imaging

**DOI:** 10.3389/fnins.2019.00903

**Published:** 2019-08-29

**Authors:** Huijun Zhang, Xuan Sun, Qiong Huang, Xiangming Wang, Yunhua Yue, Mingfeng Ju, Xiaoping Wang, Ji Ding, Zhongrong Miao

**Affiliations:** ^1^Department of Neurology, Tong Ren Hospital, Shanghai Jiao Tong University School of Medicine, Shanghai, China; ^2^Department of Interventional Neurology, Beijing Tiantan Hospital, Capital Medical University, China National Clinical Research Center for Neurological Diseases, Center of Stroke, Beijing Institute for Brain Disorders, Beijing, China; ^3^Department of Neurology, Yangpu Hospital, Tongji University School of Medicine, Shanghai, China

**Keywords:** intracranial atherosclerotic stenosis, diffusion-weighted imaging, middle cerebral artery occlusion, intracranial embolism, acute ischemic stroke

## Abstract

**Background:** The differentiation of large vessel occlusion caused by intracranial atherosclerotic stenosis (ICAS) or intracranial embolism significantly impacts the course of treatment (i.e., intravenous thrombolysis versus mechanical thrombectomy) for acute cerebral infarction. Currently, there is no objective evidence to indicate ICAS-related middle cerebral artery M1 segment occlusion before treatment. In cases of ICAS, it is often observed that the infarct core caused by ICAS-related M1 segment middle cerebral artery occlusion (MCAO) is located in deeper parts of the brain (basal ganglia or semiovoid region).

**Objective:** To evaluate whether the location of the infarct core, identified using diffusion-weighted imaging (DWI), can be used to differentiate ICAS from intracranial embolism.

**Methods:** Thirty-one consecutive patients diagnosed with acute cerebral infarction caused by middle cerebral artery M1 segment occlusion were retrospectively included based on angiographic findings to distinguish ICAS from embolic occlusion. Patients were divided into two groups based on the location of the infarct core on DWI: in the deep part of the brain (basal ganglia or semiovoid region) or more superficially (i.e., cortex).

**Results:** In 16 patients, the infarct core was mainly in the deep part of the brain on DWI [14 of 16 patients in the ICAS group and only 2 in the non-ICAS group (93.3 vs. 6.7%, respectively; *P* < 0.001)]. The diagnostic sensitivity of DWI for ICAS was 93.3%, with a specificity of 87.5%, a Positive predictive value (PPV) of 87.5%, and an Negative predictive value (NPV) of 93.3%, the accuracy was 88.5%.

**Conclusion:** Intracranial atherosclerotic disease-related acute MCAO can be predicted using DWI.

## Introduction

More than 80% of strokes are ischemic in nature, 25–35% of which result from large vessel occlusion. Patients with large vessel occlusion often experience severe neurological deficits (Kidwell et al., 2013). Without timely and appropriate treatment, these patients have a poor prognosis. Large vessel occlusions are usually caused by intracranial atherosclerotic stenosis (ICAS) or intracranial embolism. ICAS accounts for 22.9% of cases in Asia (Yoon et al., 2015).

For acute ischemic stroke caused by intracranial embolism (Berkhemer et al., 2015; Goyal et al., 2015; Jovin et al., 2015; Lee et al., 2015; Powers et al., 2015; Saver et al., 2015), mechanical thrombectomy is an effective treatment. Patients diagnosed with cerebral embolism can be directly treated with mechanical thrombectomy without intravenous thrombolysis for shortening the recanalization time. For patients with large vessel occlusion caused by ICAS, however, platelet aggregation can cause re-occlusion of the culprit vessels after mechanical thrombectomy, even if remedial measures are administered (Heo et al., 2003; Gao et al., 2015; Yoon et al., 2015). For patients in this category, a loading dose of antiplatelet agents can be used to reduce aggregation before and during surgery. Therefore, it is very important to differentiate ICAS from intracranial embolism before operating.

Intracranial atherosclerotic stenosis can be detected and assessed using high-resolution angiographic-magnetic resonance (MR) imaging (MRI) before treatment (Dieleman et al., 2014; Natori et al., 2014; Kim et al., 2015; van der Kolk et al., 2015); however, this is expensive and without definite diagnostic criteria. It can also be predicted by microcatheter “first-pass effect” during mechanical thrombectomy (Yi et al., 2018); however, there is no objective evidence to indicate intracranial atherosclerotic disease-related occlusion before surgery.

Magnetic resonance (MR) imaging plays an important role in the diagnosis and treatment of acute ischemic cerebrovascular disease. In the present study, patients with acute middle cerebral artery occlusion (MCAO) underwent diffusion-weighted imaging (DWI) to evaluate whether the location of the infarct core could be used to differentiate ICAS from intracranial embolism.

## Materials and Methods

### Patients

Thirty-one consecutive patients, who experienced acute stroke and underwent endovascular therapy between May 2017 and August 2018, were identified in the Beijing Tiantan Hospital (Beijing, China) database according to the following criteria: exhibited MCAO; time between symptom onset and admission was 6 h or >6 h for a moderate-to-large hypoperfusion area as depicted on multimodal MRI; underwent vascular recanalization, which was subsequently confirmed; age >18 years; and the prestroke modified Rankin Scale score was 0–1. Patients in whom stroke was the result of dissection, moyamoya disease or vasculitis, those with unexplained MCAO, those who did not undergo MRI before surgery or cerebrovascular examination within 1 week after the operation, and those with carotid T and carotid L collaterals were excluded. Informed consent was obtained from all participants or their relatives, and the protocol was approved by the Institutional Review Board of Beijing Tiantan Hospital.

Demographic information and patient characteristics are summarized in Table 1. The patients (18 men, 13 women) had a mean (±standard error of the mean) age of 61 ± 2 years. Seventeen (54.8%) patients were smokers. The prevalence of hypertension, diabetes, hyperlipidemia, atrial fibrillation and/or rheumatic heart disease, and transient ischemic attack in the 31 patients were 58.1, 32.3, 16.1, 32.3, and 6.45%, respectively.

**TABLE 1:** Clinical characteristics of all the patients (*n* = 31).

Sex (male, *n*)	18(58.1%)
Age	61.13(years)
Smoker	17(54.8%)
Hypertension, *n*(%)	18(58.1%)
Diabetes mellitus, *n*(%)	10(32.3%)
Hyperlipidemia, *n*(%)	5(16.1%)
Atrial fibrillation and/or rheumatic heart disease	10(32.3%)
Transient ischemic attack	2(6.45%)

### Operational Definitions of ICAS and Embolic Occlusion

**FIGURE 1:**
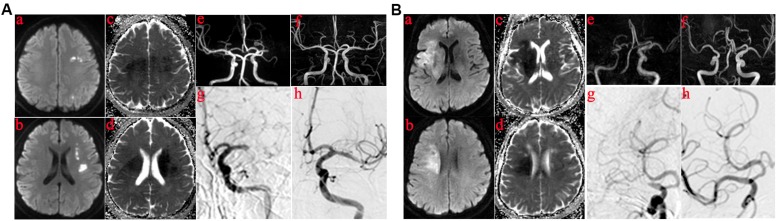
Those in whom the infarct core was mainly in the basal ganglia or semiovoid region and those in whom the infarct core was mainly in cortex. **(A)** The infarct core was mainly in basal ganglia or semiovoid region. **(a–d)** The presentation of infarcts on DWI and Apparent diffusion coefficient (ADC) mapping; **(e)** Right middle cerebral artery occlusion before operation on MRA; **(f)** MRA performed within 1 week showed severe residual stenosis in the responsibility lesions of right middle cerebral artery; **(g,h)** DSA demonstrated the results of MRA. **(B)** The infarct core was mainly in cortex. **(a–d)** The presentation of infarcts on DWI and ADC mapping; **(e)** Left middle cerebral artery occlusion before operation on MRA; **(f)** Postoperative MRA showed no residual stenosis in the responsibility lesions of right middle cerebral artery; **(g,h)** DSA demonstrated the results of MRA.

Angiographic findings distinguishing ICAS from embolic occlusion were based on those described in a previous study (Yi et al., 2018). Evidenced by final angiography or during endovascular treatment, ICAS was defined as a significant fixed focal stenosis that could be resolved using angioplasty or stent insertion (Lee et al., 2015; Yi et al., 2018) at the site of occlusion (Figures 1Ag,h). Significant stenosis was defined as fixed stenosis ≥70% or fixed stenosis ≥50%, besides either angiographically evident of impaired perfusion or evidence of re-occlusion after appropriate treatment using a stent retriever. The cause of MCAO (Figures 1Ae,Be) was classified as embolism based on the following: no focal stenosis after clot retrieval during operation (Figures 1Bg,h), and confirmed on MR angiography (Figures 1Af,Bf) or computed tomography angiography performed within 1 week after the procedure; and an embolus removed using a stent retriever.

### DWI and Clinical Assessment

Before surgery, MRI included DWI and a three-dimensional-time-of-flight-MR angiography. T1-, T2-weighted imaging, and fluid attenuated inversion recovery were performed in all patients using a 3.0 Tesla scanner (Discovery 750, GE Healthcare, Milwaukee, WI, United States) equipped with a 32-channel head coil. A single-shot echo-planar imaging DWI sequence was performed using the following parameters: repetition time/echo time, 2300/63.60 ms; b, 1000 s/mm2; slice thickness, 5 mm; slice number, 24; field of view, 240 mm; and matrix, 128 × 128. The related apparent diffusion coefficient (ADC) and exponential ADC maps were obtained. A focal hyperintensity on DWI and hypointensity on the ADC was defined as an infarct core. The site of the infarct [located deep in the brain (e.g., basal ganglia or semiovoid region, or mainly in cortex)] was investigated. DWI findings were evaluated by two neuroradiologists blinded to the clinical symptoms and surgical findings.

Neurological function in all patients was assessed on admission using the National Institutes of Health Stroke Scale (NIHSS). Patients were radiologically assessed using the Thrombolysis in Cerebral Infarction (TICI) scale, and successful reperfusion was defined as a TICI grade of 2b or 3 after endovascular treatment (Zaidat et al., 2013).

Clinical characteristics of the patients, risk factors for arteriosclerosis, heart disease, previous transient ischemic attack, NIHSS score on admission, the hyper-dense artery sign (HAS) on non-enhanced CT, and angiographic information were collected. All images were retrospectively reviewed by two neurologists blinded to patient information and study protocol; discrepancies between the reviewers were resolved by consensus discussion.

### Statistical Analysis

Differences in clinical characteristics, risk factors, and imaging features between patients in whom the infarct core was mainly in the deep part of the brain and those in whom the infarct core was mainly in the cortex were examined using bivariate analysis, as between patients with ICAS and those with intracranial embolism. The Student’s *t*-test was used to compare continuous variables, while the χ2 test was used to compare categorical variables. Diagnostic performance including sensitivity, specificity, positive predictive value (PPV), negative predictive value (NPV), and diagnostic accuracy of the location of infarct core for the prediction of ICAS, were calculated. All statistical analyses were performed using Prism version 5 (Mac OS X, Apple Inc., Cupertino, CA, United States); *P* ≤ 0.05 was considered to be statistically significant.

## Results

### Patients With ICAS vs. Patients With Embolism

Data from 15 patients diagnosed with ICAS and 16 diagnosed with embolism were compared using the χ2 test; the results are summarized in Table 2.

**TABLE 2:** Clinical characteristics and endovascular therapy of patients in the ICAS group and embolism group.

	**ICAS group**	**Embolism group**	***P*-value**
	**(*n* = 15)**	**(*n* = 16)**	
Sex (male, *n*)	9 (60%)	9 (56.2%)	1.000
Age (mean, years)	61±2	63±3	0.575
Smoker	9 (60%)	8 (50%)	0.722
Hypertension, n(%)	12 (80%)	6 (37.5%)	0.029
Diabetes mellitus, *n*(%)	5 (33.3%)	5 (31.3%)	1.000
Hyperlipidemia, *n*(%)	3 (20%)	2 (12.5%)	0.654
Atrial fibrillation and/or rheumatic			
heart disease	1 (6.67)	9 (56.2%)	0.006
Transient ischemic attack	2 (13.3)	0	0.226
Admission NHISS	12±1	15±2	0.235
HAS on CT, *n* (%)	3 (20%)	12 (75%)	0.003
With balloon or stent	12 (80%)	1 (6.25%)	<0.001
Hemorrhage	3 (20%)	7 (43.8%)	0.458
Occlusion again, *n*(%)	1 (6.67)	0 (0%)	0.484

Patients with ICAS were more likely to have hypertension (80 vs. 37.5%; *P* = 0.029), less likely to have atrial fibrillation and/or rheumatoid heart disease (6.67 vs. 56.2%; *P* = 0.006), and less likely to exhibit HAS on non-enhanced CT (20 vs. 75%, *P* = 0.003). Twelve of the 15 (80%) patients with ICAS required emergency angioplasty for successful recanalization (Table 2).

### Patients Whose Infarct Core Was Mainly in the Deep of the Brain and Those Whose Infarct Core Was Mainly in Cortex

According to DWI and ADC, patients were divided into two categories, as shown in Figures 1A (a–d). For some, the infarct core was mainly in the deep part of the brain (e.g., basal ganglia or semiovoid region) and, as shown in Figures 1B (a–d), there were some whose infarct core was mainly in the cortex, respectively. Clinical characteristics of the two patient groups are summarized in Table 3. Patients in whom the infarct core was mainly in the basal ganglia or semiovoid region group, compared with those whose infarct core mainly in cortex group, were more likely to have hypertension (80 vs. 37.5%; *P* = 0.029) and less likely to have atrial fibrillation and/or rheumatoid heart disease (6.67 vs. 56.2%; *P* = 0.006), respectively.

**TABLE 3:** Clinical characteristics of the two groups patients the infarct core were mainly in deep of the brain and cortex, respectively.

	**Deep of the brain**	**Cortex**	***P*-value**
	**(*n* = 16)**	**(*n* = 15)**	
Sex (male, *n*)	10 (62.5%)	8 (53.3%)	0.722
Age (mean, years)	60±3	65±2	0.142
Smoker	10 (60%)	7 (50%)	0.724
Hypertension, *n*(%)	12 (80%)	6 (37.5%)	0.029
Diabetes mellitus, *n*(%)	6 (37.5%)	4 (26.7%	0.704
Hyperlipidemia, *n*(%)	4 (25%)	1 (6.7%)	0.333
Atrial fibrillation and/or rheumatic			
Heart disease	1 (6.67)	9 (56.2%)	0.006
Transient ischemic attack	2 (13.3)	0	0.226

### Diagnostic Performance of the DWI

There were 16 patients in whom the infarct core was mainly in the basal ganglia or semiovoid region on DWI (14 of the 16 patients in the ICAS group and only 2 in the non-ICAS group [93.3 vs. 6.7%, respectively; *P* < 0.001]). The diagnostic sensitivity of DWI for ICAS was 93.3%, with a specificity of 87.5%, a PPV of 87.5%, and an NPV of 93.3%, the accuracy was 88.5%.

## Discussion

The purpose of our study was to explore objective evidence for discerning ICAS from intracranial embolism resulting in M1 occlusion before surgery. CT and MRI are mainly used in the diagnosis and treatment of acute cerebral infarction. However, the following advantages of MRI become necessary for examination (Bang et al., 2018). First, DWI combined with ADC is superior to any CT techniques for imaging the infarct core (Kohrmann and Schellinger, 2009). Second, mismatch between DWI and FLAIR can be used to guide intravenous recombinant tissue plasminogen activator treatment in “Wake-up” patients with acute ischemic stroke with an unknown time of onset (Thomalla et al., 2018). Finally, using MRI to assess collaterals and the infarct core can expand the scope of application of endovascular treatment (Albers et al., 2018; Nogueira et al., 2018). Thus, MRI was chosen as the main research tool for diagnosis and treatment of acute cerebral infarction.

Our data revealed no significant difference in sex or age between those with ICAS and those with embolization. One possible explanation is that our findings were based on a single-center experience and that the sample size was insufficient, or only focused on occlusion of the M1 segment of the middle cerebral artery. Consistent with a previous study (Yi et al., 2018), we also found that the ICAS patients were more likely to have hypertension (80 vs. 37.5%; *P* = 0.029), which is a risk factor for arteriosclerosis (Lee et al., 2015), less likely to have atrial fibrillation and/or rheumatoid heart disease (6.67 vs. 56.2%; *P* = 0.006) and exhibit HAS on non-enhanced CT (20 vs. 75%; *P* = 0.003), which is related to cardiac-embolic stroke (Kirchhof et al., 2003; Cho et al., 2005; Kim et al., 2008; Moftakhar et al., 2013). Twelve of the 15 (80%) patients with ICAS required emergency angioplasty for successful recanalization (Table 2).

Although DWI had been used to analyze causes of posterior cerebral artery infarction (Lee et al., 2009), there has been no research using DWI to study the characteristics of infarcts caused by MCAO of the M1 segment in ICAS. In assessing DWI results of patients with acute MCAO, we found that the infarction core of the patients with ICAS were primarily located in the deeper parts of the brain (basal ganglia and semiovoid regions), and the infarction core of occlusions caused by intracranial embolism were located more superficially (i.e., cortex). Our study revealed a significant association between the location of the infarct core detected on DWI and causes of middle cerebral artery M1 occlusion. There were 16 patients whose infarct core was mainly in the deep of the brain on DWI. The diagnostic sensitivity of DWI for ICAS was 93.3%, with a specificity of 87.5%, a PPV of 87.5%, and an NPV of 93.3%, the accuracy was 88.5%. Our study demonstrated that the infarct core in patients with ICAS was located primarily in the deeper parts of the brain, which has two possible explanations. First, in ICAS, the collateral circulation in the cortex is abundant. Second, chronic ischemia may increase the ischemic tolerance of cortical neurons. As our investigation was a single-center retrospective study, a multicenter study involving a larger sample size or a randomized controlled trial are needed to further verify our results.

## Conclusion

The features of infarction on DWI can predict MCAO caused by ICAS preoperatively, which may reflect the ischemic tolerance of cortical neurons. Increased ICAS can guide therapeutic strategies in patients with acute cerebral infarction. First, patients diagnosed with cerebral embolism can be directly treated with mechanical thrombectomy without intravenous thrombolysis for shortening the recanalization time. Second, for these patients, intravenous thrombolysis for within “time window” or dual antiplatelet loading dose for out of “time window” is a better option. Third, if diagnosed with ICAS, it should be considered preoperatively, and stent or balloon expansion therapy should be administered in time to reduce vascular injury caused by repeated thrombolysis. Finally, the correct diagnosis provides an appropriate basis for secondary prevention of stroke.

## Ethics Statement

Our access to patients’ records for data collection and analysis of the data were approved by Beijing Tiantan Hospital, Capital Medical University medical ethics committee.

## Author Contributions

HZ and XS designed the research. HZ, XS, and ZM performed the research. QH, XmW, YY, MJ, XpW, and JD analyzed the data. ZM wrote the manuscript.

## Conflict of Interest Statement

The authors declare that the research was conducted in the absence of any commercial or financial relationships that could be construed as a potential conflict of interest.

## Abbreviations

ADC: apparent diffusion coefficient
DWI: diffusion-weighted image
HAS: hyper-dense artery sign
ICAS: intracranial atherosclerotic stenosis
MCAO: middle cerebral artery occlusion
NIHSS: NIH stroke scale
NPV: negative predictive value
PPV: positive predictive value
TICI: thrombolysis in cerebral infarction.
